# *Arabidopsis thaliana* as a tool to identify traits involved in *Verticillium dahliae* biocontrol by the olive root endophyte* Pseudomonas fluorescens* PICF7

**DOI:** 10.3389/fmicb.2015.00266

**Published:** 2015-04-07

**Authors:** M. Mercedes Maldonado-González, Peter A. H. M. Bakker, Pilar Prieto, Jesús Mercado-Blanco

**Affiliations:** ^1^Department of Crop Protection, Institute for Sustainable Agriculture, Agencia Estatal Consejo Superior de Investigaciones Científicas, CórdobaSpain; ^2^Plant-Microbe Interactions, Institute of Environmental Biology, Utrecht University, UtrechtNetherlands; ^3^Department of Plant Breeding, Institute for Sustainable Agriculture, Agencia Estatal Consejo Superior de Investigaciones Científicas, CórdobaSpain

**Keywords:** *Arabidopsis*, biological control, endophyte, induced systemic resistance, olive, * Pseudomonas fluorescens* PICF7, root colonization, *Verticillium dahliae*

## Abstract

The effective management of Verticillium wilts (VW), diseases affecting many crops and caused by some species of the soil-borne fungus *Verticillium*, is problematic. The use of microbial antagonists to control these pathologies fits modern sustainable agriculture criteria. *Pseudomonas fluorescens* PICF7 is an endophytic bacterium isolated from olive roots with demonstrated ability to control VW of olive caused by the highly virulent, defoliating (D) pathotype of *Verticillium dahliae* Kleb. However, the study of the PICF7-*V. dahliae*-olive tripartite interaction poses difficulties because of the inherent characteristics of woody, long-living plants. To overcome these problems we explored the use of the model plant *Arabidopsis thaliana*. Results obtained in this study showed that: (i) olive D and non-defoliating *V. dahliae* pathotypes produce differential disease severity in *A. thaliana* plants; (ii) strain PICF7 is able to colonize and persist in the *A. thaliana* rhizosphere but is not endophytic in *Arabidopsis*; and (iii) strain PICF7 controls VW in *Arabidopsis*. Additionally, as previously observed in olive, neither swimming motility nor siderophore production by PICF7 are required for VW control in* A. thaliana*, whilst cysteine auxotrophy decreased the effectiveness of PICF7. Moreover, when applied to the roots PICF7 controlled *Botrytis cinerea* infection in the leaves of *Arabidopsis*, suggesting that this strain is able to induce systemic resistance. *A. thaliana* is therefore a suitable alternative to olive bioassays to unravel biocontrol traits involved in biological control of *V. dahliae* by *P. fluorescens* PICF7.

## Introduction

*Verticillium* spp. are soil-borne, cosmopolitan ascomycete fungi producing vascular wilts and severe losses in many economically relevant crops worldwide (Pegg and Brady, 2002; Fradin and Thomma, 2006; Inderbitzin et al., 2011). *Verticillium dahliae* Kleb. causes most of the known Verticillium wilts (VW; Agrios, 1997; Jiang et al., 2005). It seriously compromises olive (*Olea europaea* L.) cultivation in many countries of the Mediterranean Basin, producing Verticillium wilt of olive (VWO). Effective control of this disease is difficult because of a number of contributing factors (Tsror, 2011). In fact, none of the currently available measures are completely successful when applied individually. Therefore, the implementation of an integrated disease management strategy is proposed as the most effective way to control VWO, with emphasis on preventive (pre-planting) actions (López-Escudero and Mercado-Blanco, 2011). One of these measures is the use of antagonistic rhizobacteria as biological control agents (BCA), particularly in pathogen-free certified olive plants at the nursery-production stage (Tjamos, 1993). Beneficial rhizosphere *Pseudomonas* spp. strains have been extensively studied and used as BCA, exploiting a range of mechanisms including production of antibiotics, competition for nutrients and/or colonization sites, and induced systemic resistance (Weller et al., 2002; Haas and Défago, 2005; Mercado-Blanco and Bakker, 2007). Selected strains of *Pseudomonas* spp. have thus shown successful in suppressing VW in different susceptible hosts, including olive (Berg et al., 2006; Debode et al., 2007; Uppal et al., 2008; Erdogan and Benlioglu, 2010; Sanei and Razavi, 2011; Triki et al., 2012).

The olive root endophyte *P. fluorescens* PICF7 is an effective BCA of VWO caused by the defoliating (D, highly virulent) pathotype of *V. dahliae* in nursery-propagated olive plants (Mercado-Blanco et al., 2004; Prieto et al., 2009; Maldonado-González et al., 2015). Upon olive root colonization, strain PICF7 elicits a broad range of defense responses both locally (roots; Schilirò et al., 2012) and systemically (aerial organs; Gómez-Lama Cabanás et al., 2014). Results from these studies indicated that systemic defense responses, either Systemic Acquired Resistance (SAR; Durrant and Dong, 2004) and/or Induced Systemic Resistance (ISR; Bakker et al., 2007), can be triggered in olive tissues after inoculation with PICF7. SAR and ISR are phenotypically similar, leading to an enhanced resistance state of the plant. While salicylic acid (SA) plays a major role in SAR (Gaffney et al., 1993; Sticher et al., 1997), ISR works through jasmonic acid (JA) and ethylene (ET) signaling pathways in most cases (Pieterse et al., 2014), although there are exceptions (Audenaert et al., 2002). Remarkably, SA, JA, and ET pathways have undefined boundaries at some points and can show cross-talk between them (Kunkel and Brooks, 2002; Koornneef and Pieterse, 2008; Zamioudis and Pieterse, 2012). This situation has been observed in plant defense responses triggered by beneficial endophytic bacteria (Conn et al., 2008), including the interaction olive-*P. fluorescens* PICF7 (Schilirò et al., 2012; Gómez-Lama Cabanás et al., 2014). From a practical perspective, simultaneous triggering of the SA and the ET/JA signaling pathways can lead to enhanced disease suppression thereby improving implementation of biological control (Van Wees et al., 2000).

To demonstrate ISR-mediated disease suppression the BCA and the pathogen need to be spatially separated throughout the experiment to rule out direct interaction between the microorganisms (Van Loon et al., 1998). Whether ISR is involved in biocontrol of *V. dahliae* by strain PICF7 in olive is difficult to assess since both microorganisms share the same ecological niche -the root system. One possibility would be the use of a split-root system, but this approach is complicated in olive. Another possibility is to evaluate PICF7 biocontrol performance against olive pathogens affecting above-ground organs (Maldonado-González et al., 2013). Here we use the model plant *Arabidopsis thaliana,* with a range of available mutants in defense signaling pathways, to unravel the involvement of induced resistance (Segarra et al., 2009). *A. thaliana* has previously been used to evaluate efficacy of BCAs (Meschke and Schrempf, 2010), including ISR-mediated biocontrol of *V. dahliae* (Tjamos et al., 2005).

Without excluding additional mechanisms (i.e., antibiosis, competition, etc.) induction of plant defense response seems to explain the biocontrol activity exerted by strain PICF7 (Schilirò et al., 2012; Gómez-Lama Cabanás et al., 2014). However, little is known about PICF7 traits implicated in biocontrol efficacy. Mutant analysis have recently revealed that production of the siderophore pyoverdine (Pvd) and swimming motility are not required for control of VWO nor for endophytic colonization by PICF7 (Maldonado-González et al., 2015). Strain PICF7 exhibits good and prolonged surface and endophytic colonization abilities in different olive cultivars and under diverse experimental conditions (Mercado-Blanco et al., 2004; Prieto and Mercado-Blanco, 2008; Prieto et al., 2011). Moreover, colonization ability of strain PCF7 is not limited to olive root tissues since our previous studies have demonstrated that it can colonize and persist in olive stems (Maldonado-González et al., 2013) and even in the root system of sunflower (*Helianthus annuus* L.; Maldonado-González et al., 2012). Inner and rhizoplane colonization of olive roots by PICF7 seems to be crucial for VWO biocontrol efficacy of strain PICF7 (Prieto et al., 2009).

*Verticillium dahliae* isolates infecting olive have been classified into D and non-defoliating (ND, moderately virulent) pathotypes (López-Escudero and Mercado-Blanco, 2011), which correlates with their genetic and molecular differences (Mercado-Blanco et al., 2003; Collado-Romero et al., 2006). Differential virulence displayed by isolates that infect olive was shown also in cotton (*Gossypium hirsutum* L.; Schnathorst and Sibbett, 1971; Dervis et al., 2010). However, D-pathotype isolates do not behave as the most virulent group in artichoke (*Cynara scolymus* L.; Jiménez-Díaz et al., 2006). Pathogenicity of *A. thaliana* by *V. dahliae* has been demonstrated earlier (Soesanto and Termorshuizen, 2001; Veronese et al., 2003; Tjamos et al., 2005; Zhao et al., 2014). However, there is no information on whether *V. dahliae* olive D and ND pathotypes induce the same differential virulence in *A. thaliana* plants than that observed in olive and cotton.

The main objective of this study was to assess whether the model plant *A. thaliana* can be used to identify *P. fluorescens* PICF7 traits involved in the control of *V. dahliae*. To achieve this, several sub-objectives were pursued: (i) to determine whether *V. dahliae* olive pathotypes (D and ND) cause differential virulence in *A. thaliana*; (ii) to assess whether *P. fluorescens* PICF7 colonizes and persists in the root system of different *A. thaliana* genotypes; (iii) to check whether strain PICF7 is able to endophytically colonize *A. thaliana* roots; (iv) to investigate whether strain PICF7 is able to control VW in different *A. thaliana* genotypes; (v) to determine whether specific PICF7 phenotypes behave in *A. thaliana* as previously observed in olive plants; and (vi) to find out if PICF7 is able to elicit an ISR response in *A. thaliana* using the leaf pathogen *Botrytis cinerea*.

## Materials and Methods

### Bacterial Strains, Fungal Isolates, Growth Conditions and Inoculum Production

*Pseudomonas fluorescens* PICF7 (Mercado-Blanco et al., 2004; Martínez-García et al., 2015), four Tn5-Tc^R^ (tetracycline-resistant) transposon insertion mutants (Maldonado-González et al., 2015), a PICF7 fluorescently tagged derivative (Prieto and Mercado-Blanco, 2008) and a *P. fluorescens* WCS417 rifampicin-resistant spontaneous mutant (WCS417r; Lamers et al., 1988) were used in this study (**Table 1**). Strain PICF7 mutant ME424 is impaired in swimming motility, mutant ME589 lacks siderophore Pvd production, mutant ME419 shows growth delay in potato dextrose agar (PDA) medium, and mutant ME1508 is a cysteine (Cys) auxotroph (Maldonado-González et al., 2015; **Table 1**). To determine strain PICF7’s ability to colonize roots of *Arabidopsis*, a Tc^R^ enhanced green fluorescent protein (EGFP)-labeled derivative (harboring plasmid pMP4655; Bloemberg et al., 2000; Prieto and Mercado-Blanco, 2008) was used in confocal laser scanning microscopy (CLSM) experiments (see below). To evaluate possible systemic defense responses strain WCS417r was used. All bacterial strains were grown at 28°C on King’s medium B (King et al., 1954) agar (KBA) plates, when needed supplemented with antibiotics at the following concentrations (mg⋅l^-1^): tetracycline (Tc, 20); ampicillin (Amp, 50); chloramphenicol (Chl, 13); natamycin (Nat, 100) and rifampicin (Rf, 50).

**Table 1:** Bacterial strains and plasmids used.

Strains or plasmids	Characteristics	Reference
**Bacterial strains**
*P. fluorescens*		
PICF7	Wild-type olive root endophyte	Mercado-Blanco et al. (2004)
ME419	PICF7 Tn5 (Tc^R^) *in vitro* growth mutant derivative, GltA^-^	Maldonado-González et al. (2015)
ME424	PICF7 Tn5 (Tc^R^) motility mutant derivative, FliI^-^	Maldonado-González et al. (2015)
ME589	PICF7 Tn5 (Tc^R^) pyoverdine mutant derivative, PvdI^-^	Maldonado-González et al. (2015)
ME1508	PICF7 Tn5 (Tc^R^) auxotroph Cys mutant derivative	Maldonado-González et al. (2015)
PICF7 (pMP4655)	PICF7 (Tc^R^) EGFP-labeled derivative	Prieto and Mercado-Blanco (2008)
WCS417r	Spontaneous rifampicin mutant of strain WCS417	Lamers et al. (1988)
**Plasmids**
pMP4655	oriBBR1, oriVS1, oriT (p15A), *lac::eGPF*, Tc^R^	Bloemberg et al. (2000)

*GltA, Type II citrate synthase; FliI, flagellum-specific ATP synthase; PvdI, putative pyoverdine non-ribosomal peptide synthetase; Tc, tetracycline; Cys, cysteine; EGFP, Enhanced Green Fluorescence Protein*.

*Pseudomonas* inoculum was prepared as described by Maldonado-González et al. (2013). Bacterial cell densities required for each experiment were adjusted spectrophotometrically (A600 nm) by building up standard curves and culturing viable cells from serial dilution series onto KBA plates (to count PICF7 wild-type colonies), or KBA plates supplemented with Tc or Rf (for Tn5 mutant and EGFP-labeled PICF7 derivatives and WCS417r, respectively).

Four isolates of *V. dahliae*, three representative of the D pathotype [V150I and V937I isolated from olive and V138I originated from cotton, all belonging to the vegetative compatibility group (VCG) 1A] and one of the ND pathotype (V789I, belonging to VCG4B and isolated from olive; Collado-Romero et al., 2006), were used in this study. These isolates are deposited in the culture collection of the Department of Crop Protection, Institute for Sustainable Agriculture (CSIC), Córdoba, Spain. Inocula of *V. dahliae* isolates were prepared as described by Mercado-Blanco et al. (2004).

The necrotrophic fungus *B. cinerea* was used to carry out ISR assays. A conidial suspension (100 μl) of the pathogen (stored at –80°C) was inoculated on half-strength PDA plate and grown at 22°C for one month at 9.5-h photoperiod (100 μmol⋅m^-2^⋅s^-1^). Then, 5–10 ml of half-strength PDB was added to the plates, and conidia were released from the mycelium by scraping with a sterile glass rod. The conidial suspension was filtered through sterile glass wool and the density was adjusted with sterile half-strength PDB.

### Plant Material and Plant Growth Conditions

Several genotypes of *A. thaliana* were used: wild-type Col-0 and its derivatives* ein2* (ET insensitive2, affected in the protein EIN2, central component in the ET signal transduction pathway and first positive regulator in the route; Guzmán and Ecker, 1990), *jar1* (affected in jasmonyl isoleucine conjugate synthase 1, enzyme essential in the production of JA; Pieterse et al., 1998), *myb72* (affected in R2R3-MYB-like transcription factor protein, unable to elicit ISR response; Van der Ent et al., 2008), *sid1* (defective in a member of the MATE [multidrug and toxic compound extrusion transporter] family, required for SA accumulation, no SAR response; Serrano et al., 2013), and *sid2* (isochorismate synthase mutant, unable to elicit SAR response; Nawrath and Métraux, 1999). Seeds were carefully distributed over wet river sand supplemented with half-strength Hoagland nutrient solution contained in a small tray. This setup, conveniently moist, was placed within a covered tray. After 2 or 3 weeks in a growth chamber at 21 ± 1°C, 100% relative humidity and 8-h photoperiod (200 μmol⋅m^-2^⋅s^-1^), seedlings were used for colonization/biocontrol assays or pathogenicity tests. After transplanting, plant growth conditions for all experiments were 21 ± 1°C, 70 % relative humidity and 8-h photoperiod of fluorescent light as indicated above.

### Verticillium Wilt Development in *A. thaliana*: Pathogenicity Tests

To determine whether olive- and cotton-infecting *V. dahliae* isolates produce disease symptoms in *A. thaliana* plants, isolates V138I, V150I, V937I, and V789I (see above), were tested in accession Col-0 and its mutant derivatives *ein2*, *jar1*, and *sid1* (see above). Three-week-old *Arabidopsis* plants (20) of each genotype were inoculated by dipping their root system in a conidial suspension (7.5⋅10^5^–2.9⋅10^6^ conidia/ml) of each *V. dahliae* isolate or in distilled sterile water (control treatment). *Arabidopsis* seedlings were then gently transplanted to soil (potting soil:river sand, 12:5) previously autoclaved twice for 20 min with a 24-h interval. Plants were grown in a growth chamber under controlled conditions described above. Disease incidence (*DI*) was scored as the percentage of diseased leaves of the total number of leaves infection according to the following scale: 0, no symptom; 1, 1–33%; 2, 34–66%; 3, 67–100%; and 4, dead plant. Disease score was performed twice a week after pathogen inoculation during the first month and, onward, every 7 days [14, 18, 21, 25, 32 and 39 days after inoculation DAI)].

Data were submitted to analysis of variance (ANOVA). Disease severity data were used to calculate: (i) a disease intensity index (*DII*) defined as *DII* = (Σ*Si* ×*Ni*)/(4 ×*Nt*), where *Si* is severity of symptoms, *Ni* is the number of plants with *Si* symptoms severity, and *Nt* the total number of plants; (ii) the final *DI* determined as the percentage of affected plants; and (iii) the standardized area under the disease progress curve (*SAUDPC*) of *DII* plotted over time (days; Campbell and Madden, 1990). ANOVA was calculated by means of Statistix (NH Analytical Software, Roseville, MN, USA). Treatment means were compared using Fisher’s protected least significant difference (LSD) test at α = 0.05.

### Colonization of *A. thaliana* Rhizosphere by *P. fluorescens* PICF7 and Its Mutant Derivatives

To demonstrate whether strain PICF7 is capable to colonize and persist in the rhizosphere/roots of *A. thaliana* plants, three *Arabidopsis* genotypes where used (Col-0 and its mutant derivatives *myb*72 and *sid*2). Colonization ability of PICF7 mutants ME419, ME424, ME589, and ME1508 (**Table 1**) was also evaluated in Col-0 plants. Prior to transplantation of 2-week-old *Arabidopsis* seedlings, the soil (potting soil:river sand, 12:5) was bacterized with a cells suspension of PICF7 or each mutant derivative (8.0⋅10^7^–2.8⋅10^8^ cfu/g soil) as described by Djavaheri et al. (2012). Plants were kept in a growth chamber (conditions describe above) for 3–4 additional weeks. Then, three root systems per bacterial treatment were harvested and shaken for 1 min in 5 ml of 10 mM MgSO_4⋅_7H_2_O containing 0.5 g of glass beads (Pieterse et al., 1996). For bacteria counts, 10 μl-drops from serial dilutions of root macerates were deposited onto the surface of KBA plates (two per dilution) supplemented with Amp, Chl and Nat for PICF7, plus Tc in the case of Tc^R^ mutant derivatives. Bacterial colonies were scored after incubation at 28°C during 24 h. This experiment was performed three times. Data were subjected to ANOVA and means were compared to colonization of strain PICF7 in Col-0 plants using Two-sided Dunnett’s Multiple Comparisons with a Control at α = 0.05.

### Verticillium Wilt of *A. thaliana* Biocontrol Experiments

Bioassays were carried out to evaluate the ability of *P. fluorescens* PICF7 to control *V. dahliae* in *A. thaliana*. Likewise, the biocontrol performance of selected PICF7 mutants (ME419, ME424, ME589, and ME1508) was also tested. To assess whether *P. fluorescens* PICF7 and its mutants exerted biocontrol against *V. dahliae*, *A. thaliana* Col-0, and its mutants *myb72* (no ISR response) and* sid2* (no SAR response) were used. Two-week-old seedlings were transplanted to *Pseudomonas*-bacterized soil (potting soil:river sand, 12:5; 8.0⋅10^7^–2.8⋅10^8^cfu/g soil) or 10 mM MgSO_4_⋅7H_2_O (control). Seedlings were grown for 1 week under controlled conditions as previously mentioned. After that, plants (20–25) were uprooted, rinsed with tap water and their root systems dipped in a conidial suspension (3.9⋅10^5^–4⋅10^6^ conidia/ml) of the olive D isolate *V. dahliae* V937I. Control plants (10) were immersed in sterile distilled water.

Disease symptoms (chlorosis, wilting) were scored along the experiments twice a week according to the scale ranged from 0 to 4 previously described, and *SAUDPC*, *DII*, and final *DI* were calculated (see above). Biocontrol bioassays were repeated three (for PICF7 evaluation) or two (for mutant derivatives assessment) times. *SAUDPC* data were subjected to ANOVA and means were compared using Fisher’s protected LSD test at α = 0.05.

### *Botrytis cinerea* ISR Bioassay

To assess whether *P. fluorescens* PICF7 was able to elicit ISR in *A. thaliana* the foliar pathogen *B. cinerea* was used. Two independent bioassays were carried out with *A. thaliana* Col-0 plants. Bioassays were accomplished using 7-week-old *Arabidopsis* plants (20) previously grown either in control soil or in *P. fluorescens* PICF7- or WCS417r- (positive control) treated soil (9⋅10^7^–2⋅10^8^ cfu/g soil) for 5 weeks. Then, six to eight well-developed leaves were inoculated by applying 5-μl droplets of a conidial suspension of *B. cinerea* (1.7–7.5⋅10^5^ conidia/ml half strength PDB; Djavaheri, 2007). Plants were then kept at 100% relative humidity for 2 to 4 days and disease symptoms scored according to the following scale: 0, no symptoms; 1, small non-spreading lesion; 2, small non-spreading lesion with chlorosis; 3, spreading lesion with chlorosis; 4, spreading lesion and leaf completely chlorotic, or dead. Severity data were used to calculate percentage of disease leaves per plant and then subjected to ANOVA. Data means were compared using Fisher’s protected LSD test at α = 0.05.

### Confocal Laser Scanning Microscopy

In order to assess the colonization ability of *P. fluorescens* PICF7 in* A. thaliana* roots, experiments using *in vitro*- and pot-grown *Arabidopsis* plants (Col-0, *myb72*, and *sid2*) were conducted. The experiment with *in vitro*-propagated plants was performed using seeds of each *A. thaliana* genotype dipped in 500 μl of an EGFP-tagged PICF7 derivative (**Table 1**) bacterial suspension (1.3⋅10^9^ cfu/ml), contained in microfuge tubes, and incubated at 25°C, 400 rpm for 4 h. Then, bacterized seeds (20) of each genotype were placed separately in two different lines (10 per line) on the surface of a square water-agar plate (12 cm × 12 cm). All plates were kept in a growth chamber at 23 ± 2°C in the dark. In experiment with plants grown in pots, sterile mixed soil (potting soil:river sand, 12:5) supplemented with half-strength Hoagland solution (70 ml⋅Kg^-1^) was inoculated with the EGFP-tagged PICF7 derivative (8.4⋅10^8^ cfu/ml) and placed into pots. Then, six 2-weeks-old plants per genotype were placed individually per pot and incubated in a growth chamber at 23 ± 2°C with a 8-h photoperiod of fluorescent light (65 μmol⋅m^-2^⋅s^-1^), 100% relative humidity.

To visualize EGFP-tagged PICF7 cells, two plants per genotype were removed from the corresponding substrates (water agar or mixed soil) and the aerial part excised. In the case of seedlings explanted from pots, roots were carefully rinsed with water to eliminate soil particles. Then, fresh and intact roots were visualized under Axioskop 2 MOT microscope (Carl Zeiss, Jena GmbH, Germany) set with a krypton and an argon laser, controlled by Carl Zeiss Laser Scanning System LSM5 PASCAL software (Carl Zeiss) at time points 12, 15, 18, 29 days after bacterization (DAB) for the *in vitro* assay and 25 DAB (final time) for the *in planta* experiment. CLSM captures were transferred for analysis to Zeiss LSM Image Browser version 4.0 (Carl Zeiss). Processing of images was carried out by AdobePhotoshop CS version 8.0.1 software (Adobe Systems, San Jose, CA, USA).

## Results

### *Verticillium dahliae* Olive D and ND Pathotypes are Differentially Virulent on *A. thaliana*

**FIGURE 1:**
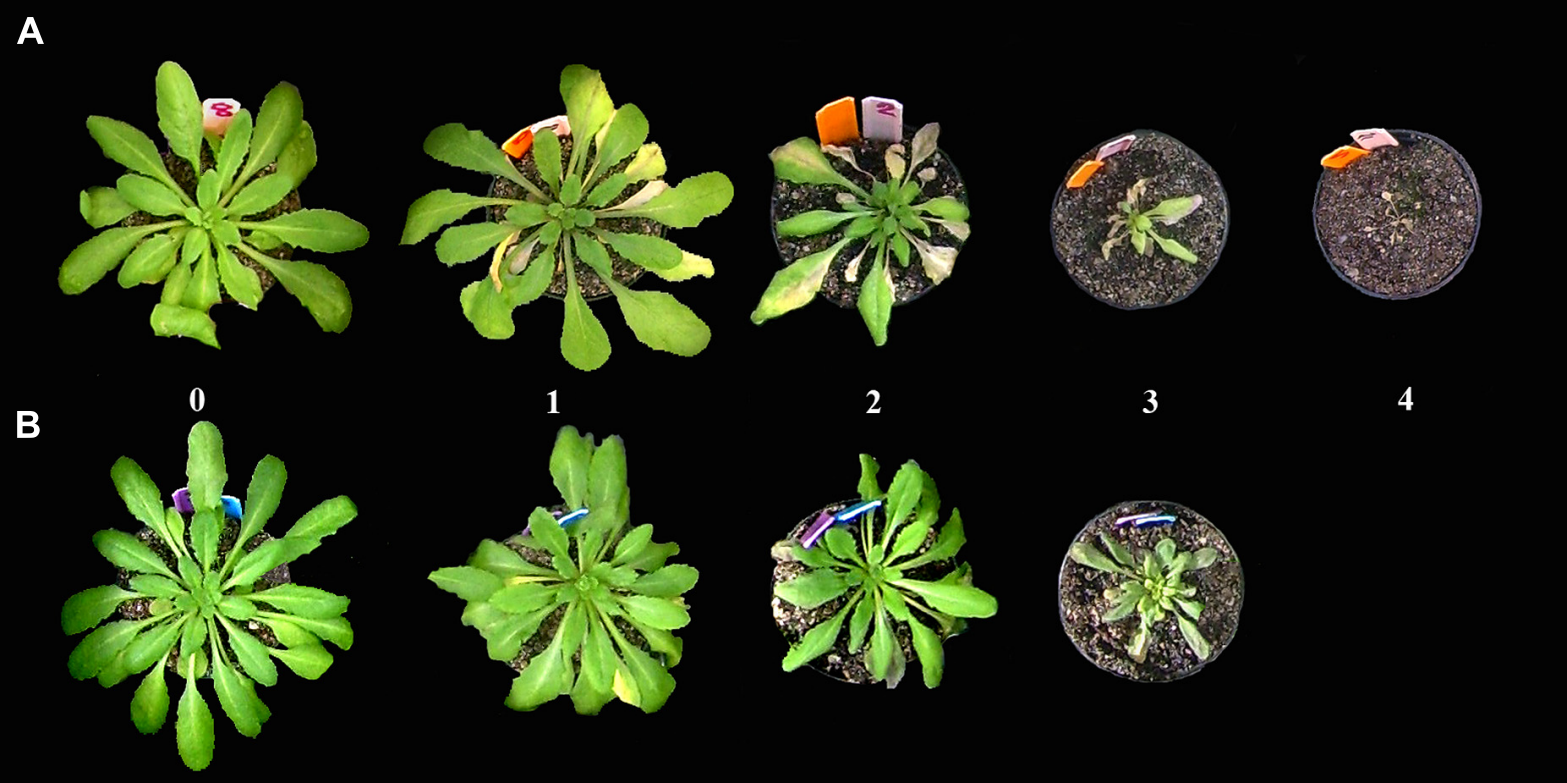
**Scale of symptoms (chlorosis, wilting) produced by the defoliating isolate V937I **(A)** and the non-defoliating (ND) isolate V789I **(B)** of *Verticillium dahliae* in* Arabidopsis thaliana* Col-0 plants**. Numbers represent the percentage of diseased leaves of the total number of leaves: 0, no symptom; 1, 1–33%; 2, 34–66%; 3, 67–100%; and 4, dead plant. Severity of symptoms produced by isolate V937I (0–4; **A**) was always higher than that observed for V789I-inoculated plants (0–3; **B**). These symptoms were observed in all *A. thaliana* genotypes analyzed in this study (see text for details).

Pathogenicity test carried out in *A. thaliana* Col-0 and its mutant derivatives *ein2* and *jar1*, insensitive to ET and JA, respectively, and *sid1* impaired in SA biosynthesis showed that all *V. dahliae* isolates tested produced VW symptoms in all *A. thaliana* genotypes. Interestingly enough, differences in symptoms appearance and severity (chlorosis, wilting and growth delay; **Figure 1**) were found depending on the infecting pathotype. Overall, disease symptoms developed earlier in plants inoculated with D isolates (V138I, V150I, and V937I) compared to ND-inoculated plants (isolate V789I; first symptoms observed at 7 and 14 DAI, respectively). Thus, severe to moderate disease symptoms were observed in all *A. thaliana* genotypes when inoculated with D isolates (*SAUPDC* values ranged from 0.43 to 0.86; *DII* 0.55 to 0.96; Final *DII* 85 to 100%; **Table 2**). In contrast, plants inoculated with isolate V789I (ND) always showed lower disease parameters (i.e., *SAUPDC* values varied from 0.23 to 0.48; *DII* 0.19 to 0.41; Final *DII* 50 to 95%) than D isolates (**Table 2**). Disease symptoms produced by *V. dahliae* V937I (D) were intermediate and, for instance, *SAUDPC* data were not significantly different from that of V789I (ND) in both Col-0 and *sid1* plants (**Table 2**). Disease severity caused by *V. dahliae* isolates was also different depending on the *A. thaliana* genotype tested. Thus, disease symptoms (*SAUDPC*) produced by isolate V150I (D) were significantly (*P* < 0.05) less severe in *jar1* than in Col-0 and *ein2* plants (**Table 2)**; isolate V937I (D) induced significantly (*P* < 0.05) higher disease severity in *ein2* plants (*SAUDPC* 0.75) than in the other tested genotypes; or isolate V789I (ND) was significantly (*P* < 0.05) less virulent in *jar1* in comparison to Col-0 and *sid2* plants (**Table 2**). Overall, V138I and V150I behaved as the most virulent isolates in all *A. thaliana* genotypes tested (**Table 2**).

**Table 2:** Pathogenicity test of selected isolates of olive defoliating (D) and non-defoliating (ND) *Verticillium dahliae* pathotypes carried out in different *Arabidopsis thaliana* accessions.

*A. thaliana* genotype	*V. dahliae* isolate	Disease assessment		
		*SAUDPC*	*DII*	Final* DI* (%)
Col-0	V138I (D)	0.80^a^	0.95	100
	V150I (D)	0.86^a^	0.95	100
	V937I (D)	0.53^cd^	0.70	100
	V789I (ND)	0.48^de^	0.39	95
				
*ein2*	V138I (D)	0.80^a^	0.94	100
	V150I (D)	0.83^a^	0.95	100
	V937I (D)	0.75^ab^	0.87	100
	V789I (ND)	0.36^ef^	0.24	80
				
*jar1*	V138I (D)	0.74^ab^	0.89	95
	V150I (D)	0.65^bc^	0.74	95
	V937I (D)	0.43^d^	0.55	85
	V789I (ND)	0.23^f^	0.19	50
				
*sid1*	V138I (D)	0.85^a^	0.96	100
	V150I (D)	0.74^ab^	0.93	100
	V937I (D)	0.49^d^	0.65	85
	V789I (ND)	0.44^d^	0.41	85

*Standardized area under the disease progress curve (SAUDPC) of a DII (disease intensity index) plotted over time and final disease incidence (*DI*, percentage of affected plants) were calculated at the end of the experiment (38 days after pathogen inoculation). Means in a column followed by different letters are significantly different in accordance to Fisher’s protected LSD test (*P* < 0.05). See Section “Materials and Methods” for details*.

### *Pseudomonas fluorescens* PICF7 Colonizes and Persists on *A. thaliana* Roots but is Not Endophytic

Strain PICF7 was able to colonize and persist on roots of Col-0, *myb72*, and *sid2* plants as bacterial counts shown after 32–40 DAB in three experiments carried out. PICF7 population sizes observed (**Table 3**) were not significantly different (*P* = 0.10; *P* = 0.64, and *P* = 0.95, respectively) among *A. thaliana* genotypes analyzed. Population sizes of native rhizobacteria found in control treatment plants were always significantly (*P* < 0.05) lower than PICF7 population sizes found in PICF7-bacterized plants but for *sid2* plants in Experiment 1. Native bacteria seemed to be displaced by introduced PICF7 cells since population sizes of the former in PICF7-treated plants were negligible and/or impossible to determine (**Table 3**).

**Table 3:** *Pseudomonas fluorescens* PICF7 root colonization ability and biocontrol performance of *V. dahliae* in different *A. thaliana* accessions.

Experiment	*A. thaliana genotype*	Treatment	Disease assessment^3^			Bacterial population (log_10_ cfu g^-1^ of fresh root)^4^	
			SAUDPC	DII	Final DI %	PICF7	Native
1	Col-0	Control	0.56^a^	0.75	94.44	na	5.0^d^
		*P. fluorescens* PICF7	0.25^b^	0.51	76.47	6.6 ± 0.1^ab^	–
	*myb*72	Control	0.49^a^	0.74	77.78	na	5.7 ± 0.3^c^
		*P. fluorescens* PICF7	0.18^bc^	0.33	44.44	6.3 ± 0.3^b^	–
	*sid*2	Control	0.24^b^	0.35	38.89	na	6.5 ± 0.4^ab^
		*P. fluorescens* PICF7	0.02^c^	0.03	5.88	6.9 ± 0.3^a^	–
					
2	Col-0	Control	0.39^abc^	0.55	80.00	na	3.7 ± 0.0^b^
		*P. fluorescens* PICF7	0.33^bc^	0.38	79.17	5.6 ± 0.2^a^	–
	*myb*72	Control	0.54^a^	0.74	92.00	na	2.8 ± 0.8^c^
		*P. fluorescens* PICF7	0.43^ab^	0.56	72.00	5.3 ± 0.4^a^	–
	*sid*2	Control	0.27^c^	0.37	68.00	na	2.9 ± 0.2^bc^
		*P. fluorescens* PICF7	0.33^bc^	0.47	72.00	5.5 ± 0.2 a	–
						
3	Col-0	Control	0.17^b^	0.27	58.82	na	4.3 ± 0.1^c^
		*P. fluorescens* PICF7	0.14^b^	0.27	72.22	7.2 ± 0.4^a^	–
	*myb*72	Control	0.14^b^	0.29	55.00	na	5.9 ± 0.5^b^
		*P. fluorescens* PICF7	0.12^b^	0.27	60.00	7.0 ± 0.4 a	–
	*sid*2	Control	0.37^a^	0.64	77.78	na	5.6 ± 0.4^b^
		*P. fluorescens* PICF7	0.22^bc^	0.43	63.16	7.0 ± 0.1 a	–
						
4	Col-0	Control	0.12^b^	0.22	47.37	na	nd
		*P. fluorescens* PICF7	0.12^b^	0.19	23.81	nd	nd
	*myb*72	Control	0.27^ab^	0.35	47.37	na	nd
		*P. fluorescens* PICF7	0.14^b^	0.23	40.00	nd	nd
	*sid*2	Control	0.39^a^	0.55	70.00	na	nd
		*P. fluorescens* PICF7	0.15^b^	0.27	42.11	nd	nd

*^1^Four independent experiments were conducted spanning 32 DAI (Experiment 1), 40 DAI (Experiments 2 and 3) or 37 DAI (Experiment 4).*

^2^The root system of 2-week-old *Arabidopsis* seedlings were transplanted into PICF7-bacterized soil (8.0*10^7^–2.8*10^8^ cfu/g soil) previously autoclaved for colonization assays. In biocontrol experiments, 1 week after PICF7-treatment plants were uprooted and their root systems dipped in a *V. dahliae* isolate V937I (D pathotype) conidia suspension (3.9·10^5^–4·10^6^ conidia/ml) or distilled sterile water (control treatment) for 15 min. Plants were then grown in a growth chamber under controlled conditions. See Section “Materials and Methods” for details.

*^3^Standardized area under the disease progress curve of *DII* plotted over time. Final *DI*, (percentage of affected plants at the end of the assay). Means in a column followed by different letters are significantly different in accordance to Fisher's protected LSD test (*P* < 0.05).*

*^4^Strain PICF7 and native bacteria cells counts were carried out on modified King’s medium B agar. Data are means of three root samples (1 g each). Means followed by different letters are significantly different in accordance to Two-sided Dunnett’s Multiple Comparisons with a Control (Col-0 plants) at α = 0.05. na, not applicable; nd, not determined in this experiment; –, native population not detected or negligible in PICF7-bacterized plants.*

**FIGURE 2:**
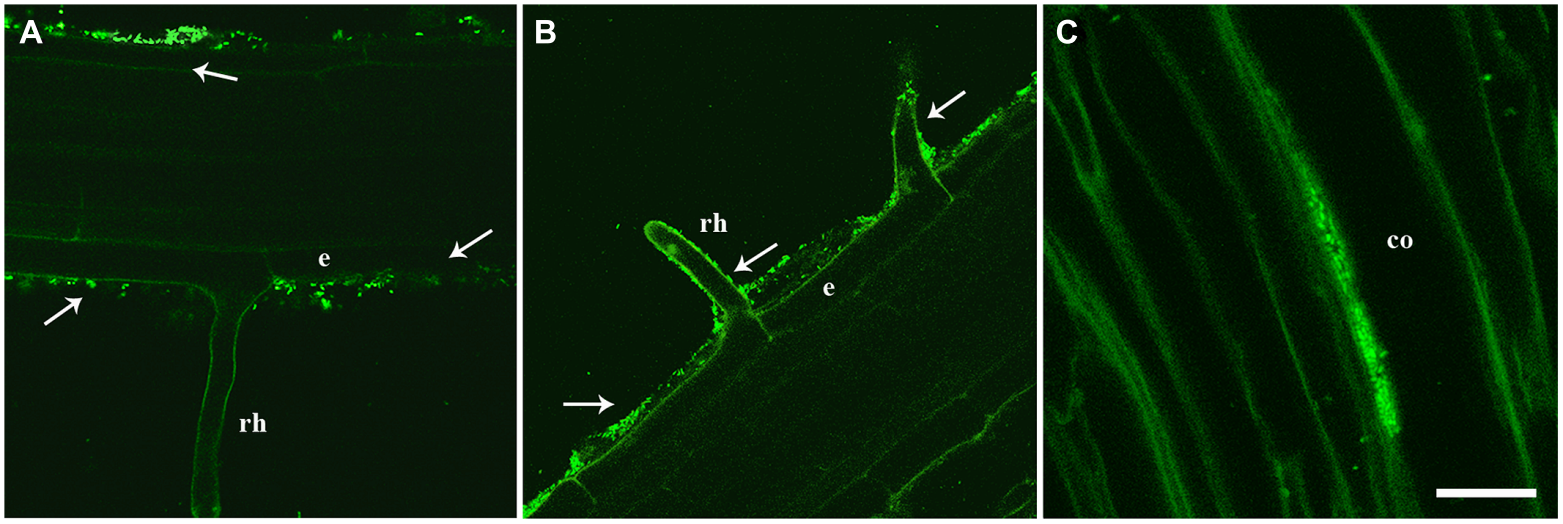
**Confocal laser scanning microscopy (CLSM) images of intact roots from two *A. thaliana* genotypes [Col-0 **(A)** and *myb72***(B)**] seedlings showing *Pseudomonas fluorescens* PICF7 (enhanced green fluorescent protein-labeled, EGFP-labeled) cells location**. Microphotographs show that PICF7 successfully colonizes the root surface of *Arabidopsis* (white arrows) but not the root interior. For comparison purposes, endophytic colonization of the root cortex of olive by PICF7 is also shown in **C**, (see Prieto and Mercado-Blanco (2008) for technical details). CLSM images were taken approximately 2 weeks after seed **(A,B)** or root-dip **(C)** bacterization with strain PICF7. Scale bar represents 50 μm in **(A**,**B)**; and 15 μm in **(C)**; co, cortical cells; e, epidermis; rh, root hair; vt, vascular tissue.

In order to assess the ability of strain PICF7 to endophytically colonize *A. thaliana* plants, roots of plants from different genotypes (Col-0, *myb72*, and *sid2*), bacterized with an EGFP-labeled PICF7 derivative, and grown either on water agar or in soil (pots) conditions were analyze by CLSM. Root samples were visualized by CLSM at 12, 15, 18, and 29 DAB (water agar) or at 25 DAB (soil). Under these experimental conditions, evidence of endophytic colonization of root tissues was not found for any of the examined *A. thaliana* genotypes, nor at any observation time. However, the rhizoplane of bacterized plants was profusely colonized by PICF7 cells (**Figures 2A,B**). In contrast, PICF7 is able to colonize the intercellular spaces of the olive root cortex (Prieto and Mercado-Blanco, 2008) as shown for comparative purpose in **Figure 2C**.

### *Pseudomonas fluorescens* PICF7 Decreases Verticillium Wilt Symptoms in *A. thaliana*

To determine whether strain PICF7 is able to control *V. dahliae* in *Arabidopsis* bioassays were conducted in which both the pathogen (isolate V937I, D pathotype) and the BCA were root inoculated. Results from four independent experiments indicated that strain PICF7 showed a trend to suppress the disease, although results varied among bioassays (**Table 3**). Thus, PICF7 was able to significantly (*P* < 0.05) suppress the disease in all *A. thaliana* genotypes (Col-0, *myb72*, and *sid2*) assessed in Experiment 1 (**Table 3**). VW control was more consistently observed in *sid2* plants (Experiments 1, 3, and 4) as revealed by *SAUDPC* values significantly (*P* < 0.05) lower in PICF7-bacterized plants compared to non-treated plants. Similarly, *DII* and final *DI* values were lower in these bioassays, but not in Experiment 3 (**Table 3**). Overall, disease parameters observed in experiments were lower in PICF7-treated plants compared to that in non-bacterized (control) plants, although differences were not statistically significant except for the cases mentioned above (**Table 3**). For instance, presence of PICF7 in *myb72* roots/rhizosphere produced a substantial decrease in all disease parameters analyzed in Experiments 2 and 4 (**Table 3**).

### Behavior of *P. fluorescens* PICF7 Mutants in *A. thaliana*

To assess whether *A. thaliana* can be used in the evaluation of PICF7 traits potentially involved in the biocontrol of *V. dahliae*, selected PICF7 mutants impaired in swimming motility or Pvd production, altered growth on PDA medium, or displaying Cys auxotrophy were used in three independent bioassays. Overall, results showed that neither swimming motility nor Pvd production are implicated in the effective biocontrol of VW in *Arabidopsis* by strain PICF7, in contrast to mutations affecting its growth in PDA (mutant ME419) or Cys auxotrophy (mutant ME1508; **Table 4**). Differences in biocontrol performance were found among experiments, though. For instance, ME1508-bacterized (Experiment 1) and ME419-treated (Experiment 2) plants showed a significant (*P* < 0.05) increase in *SAUDPC* compared to plants pre-treated with the parental strain PICF7 (**Table 4**). Furthermore, all PICF7 mutants displayed higher *DII* values than that scored for strain PICF7 in bioassays I and II (**Table 4**). Results from bioassay III did not show any significant difference among treatments. However, higher final *DI* percentages were observed for all mutant treatments compared to PICF7-treated plants (**Table 4**). Low disease pressure in this bioassay compared to that scored in the other two experiments could explain lack of significant differences. Finally, all bacterial strains colonized and persisted on roots of Col-0 plants. Nevertheless, some differences were found among experiments. Thus, mutant population sizes of all strains were not significantly different at the end of Experiments 1 and 2. Nonetheless, PICF7 showed a significantly (*P* < 0.05) larger population size in Experiment 3. Interestingly, mutant ME1508 (Cys auxotrophy) showed the lowest population size in all experiments (**Table 4**).

**Table 4:** Root colonization ability and *V. dahliae* biocontrol performance of *P. fluorescens* PICF7 and their mutant derivatives in Col-0 *A. thaliana* plants.

Experiment^1^	Treatment^2^	Disease assessment^3^			Bacterial population (log_10_ cfu g^-1^ of fresh root)^4^
		*SAUDPC*	*DII*	Final DI (%)	
1	Control	0.37^ab^	0.61	73.08	nd
	*P. fluorescens*				
	PICF7	0.25^b^	0.41	60.00	5.7 ± 0.2
	ME419 (*gltA*)	0.33^ab^	0.60	79.17	5.5 ± 0.3
	ME424 (*fliI*)	0.35^ab^	0.63	79.17	5.6 ± 0.1
	ME589 (*pvdI*)	0.35^ab^	0.50	65.38	5.7 ± 0.3
	ME1508 (Cys Aux)	0.45^a^	0.65	84.00	5.3 ± 0.2
					
2	Control	0.18^ab^	0.27	58.82	nd
	*P. fluorescens*				
	PICF7	0.15^b^	0.27	72.22	7.2 ± 0.4
	ME419 (*gltA*)	0.36^a^	0.55	84.00	6.6 ± 0.3
	ME424 (*fliI*)	0.25^ab^	0.44	66.67	7.2 ± 0.2
	ME589 (*pvdI*)	0.28^ab^	0.44	68.00	7.0 ± 0.4
	ME1508 (Cys Aux)	0.33^ab^	0.50	65.22	6.4 ± 0.2
					
3	Control	0.13^a^	0.22	47.37	nd
	*P. fluorescens*				
	PICF7	0.12^a^	0.19	23.81	7.5 ± 0.4
	ME419 (*gltA*)	0.11^a^	0.20	36.36	6.5 ± 0.2^∗^
	ME424 (*fliI*)	0.22^a^	0.31	40.00	6.4 ± 0.1^∗^
	ME589 (*pvdI*)	0.22^a^	0.43	56.00	6.8 ± 0.4^∗^
	ME1508 (Cys Aux)	0.14^a^	0.24	36.00	6.0 ± 0.4^∗^

*^1^Three independent experiments were performed. Experiments 1 and 2 spanned 36 days and Experiment 2 spanned 39 days*.

*^2^ The root system of 2-week-old *Arabidopsis* seedlings were transplanted into PICF7 or Tc^R^-mutant derivatives bacterized soil (1.0–2.8⋅10^8^ cfu/g soil) previously autoclaved for colonization experiment. For biocontrol assays, 1 week after bacterization plants were uprooted and their roots dipped in a *V. dahliae* isolate V937I (D pathotype) conidia suspension (3.9⋅10^5^–1.7⋅10^6^ conidia/ml) or distilled sterile water (control treatment) for 15 min. Plants were grown in a growth chamber under controlled conditions. See Section “Materials and Methods” for details*.

*^3^Standardized area under the disease progress curve of *DII* plotted over time. Final *DI* (percentage of affected plants at the end of the experiment). Means in a column followed by different letters are significantly different in accordance to Fisher’s protected LSD test (*P* < 0.05)*.

*^4^*Pseudomonas* cell counts were conducted on modified King’s medium B agar. Data are means of three root samples (1 g each). Means followed by an asterisk are significantly different in accordance to Two-sided Dunnett’s Multiple Comparisons with a Control (PICF7) at α = 0.05. nd, not applicable*.

### *Pseudomonas fluorescens* PICF7 Elicits Systemic Defense Responses Against *B. cinerea* in *A. thaliana*

To determine whether *P. fluorescens* PICF7 can elicit systemic defense responses in aerial tissues upon colonization of the root system, disease development by *B. cinerea* inoculated on the leaf was determined in Col-0 plants in two independent experiments. Results showed that presence of strain PICF7 in roots reduced *DI* caused by *B. cinerea* in Col-0 plants in both experiments and to the same extent as strain WCS417r, although this decrease was significant (*P* < 0.05) only in Experiment 2 (**Table 5**).

**Table 5:** *Botrytis cinerea* biocontrol by* P. fluorescens* PICF7 and WCS417r in *A. thaliana* plants.

Experiment^1^	Treatment^2^	Diseased leaves (%)^3^
1	Control	60.18^a^
	WCS417r	50.00^b^
	PICF7	58.55^ab^
		
2	Control	76.82^a^
	WCS417r	69.57^ab^
	PICF7	66.90^b^

*^1^Two independent experiments were carried spanning 7 weeks*.

*^2^Two-week-old *Arabidopsis* Col-0 seedlings were transplanted into control soil or PICF7- or WCS417r- bacterized soil (9⋅10^7^–2⋅10^8^ cfu/g soil) previously autoclaved. Five weeks later, six to eight well-developed leaves were inoculated by applying 5-μl droplets of a conidial suspension of *B. cinerea* (1.7–7.5⋅10^5^ conidia/ml half strength PDB). Plants were grown in growth chamber under controlled conditions*.

*^3^Percentage of diseased leaves. Means followed by different letters are significantly different in accordance to Fisher’s protected LSD test (*P* < 0.05)*.

## Discussion

Control of VWO is difficult, encouraging the implementation of an integrated disease management strategy (López-Escudero and Mercado-Blanco, 2011; Tsror, 2011). The use of microbial antagonists is gaining attention as an environmentally friendly approach for VWO control, particularly as a preventive measure (Mercado-Blanco and López-Escudero, 2012). Previous studies have shed light on potential mechanisms of *P. fluorescens* PICF7 involved in *V. dahliae* control and the endophytic lifestyle this bacterium shows in olive roots (Prieto et al., 2009; Schilirò et al., 2012; Gómez-Lama Cabanás et al., 2014; Maldonado-González et al., 2015). However, traits responsible for the successful biocontrol of VWO exerted by PICF7 remain mostly unknown. Furthermore, they are very complex to elucidate because of, among other factors, the idiosyncrasy of the host plant (i.e., longevity, large size, long duration of bioassays, lack of mutants, etc.). Therefore, the present research aimed to explore whether the short-living, genetically well known, and easy-to-manipulate model plant *A. thaliana* was amenable to facilitate and expedite the search for strain PICF7 traits implicated in VW suppression, and whether results obtained with this model system are similar to that observed in the natural tripartite interaction olive-*P. fluorescens* PICF7-*V. dahliae*.

*Verticillium dahliae* isolates infecting olive (and cotton) are classified into D and ND pathotypes, the former being generally more virulent than the latter (Schnathorst and Mathre, 1966; Mercado-Blanco et al., 2003; López-Escudero et al., 2004). Nevertheless, a complete correspondence between molecular/genetic/pathogenic groups (Collado-Romero et al., 2006) is not always found, and a continuum of virulence has been reported (Dervis et al., 2010). Furthermore, they can differ in pathogenicity and virulence depending on the host (Jiménez-Díaz et al., 2006). Since we aimed to assess whether *A. thaliana* can be used for the evaluation of the VWO biocontrol performance of *P. fluorescens* PICF7, it was necessary to determine the pathogenicity and virulence of selected D and ND in this model plant. Results showed that all D isolates originating from cotton (V138I) or olive (V150I and V937I) used in this study caused more severe disease symptoms than the olive ND pathotype (V789I). Therefore, virulence displayed by *V. dahliae* isolates in *Arabidopsis* plants correlated to that observed in olive. Interestingly, isolate V937I had an intermediate virulence and no difference was found between this D representative and isolate V789I in Col-0 and *sid1* plants, suggesting that the continuum of virulence previously observed in olive (Dervis et al., 2010) is also found in *Arabidopsis*. The fact that ND and D olive isolates were pathogenic in *Arabidopsis* and that both pathotypes showed the same differential virulence in this host and in olive meant that the first objective of our study was accomplished. In order to avoid excessive disease pressure that could potentially mask disease suppression effectiveness by the BCA, isolate V937I was selected for subsequent biocontrol experiments.

Efficient colonization of the target plant tissue by a BCA is a prerequisite for effective biocontrol (Lugtenberg et al., 2001; Mercado-Blanco and Bakker, 2007). Furthermore, endophytic lifestyle displayed by some rhizobacteria leading to benefits to the plant is an interesting biotechnological potential to be explored (Mercado-Blanco and Lugtenberg, 2014). The biocontrol strain PICF7 colonizes and persists on/in olive root tissues (Mercado-Blanco et al., 2004; Prieto and Mercado-Blanco, 2008). It can also persist in olive stems after artificial inoculation (Maldonado-González et al., 2013), and it efficiently colonizes the root system of an unrelated species such as sunflower (Maldonado-González et al., 2012). Results from this present study demonstrated that strain PICF7 is also able to colonize and persist on roots of *A. thaliana* genotypes, indicating that this BCA has a wide host colonization range. This apparent broad colonization ability makes it strain PICF7 as an excellent candidate to be studied as a model bacterium in plant–microbe interactions. However, no evidence of endophytic colonization was found under experimental conditions used. Indeed, while PICF7 is able to internally colonize the root hairs (Prieto et al., 2011), the intercellular spaces of the root cortex (Prieto and Mercado-Blanco, 2008; **Figure 2C**), and the root vascular tissue (Maldonado-González et al., 2015) of olive, endophytic lifestyle of PICF7 seems to be hindered in *Arabidopsis* (**Figures 2A,B**).

*Pseudomonas fluorescens* PICF7 is able to induce a multiplicity of defense responses in olive root tissues upon root inoculation (Schilirò et al., 2012). Recently, defense responses were shown to be also induced systemically, and it has been hypothesized that both SA- and JA/ET-mediated signaling responses can be involved in biocontrol exerted by PICF7 (Gómez-Lama Cabanás et al., 2014). However, actual implication of ISR and/or SAR responses in suppression of VWO has not yet been demonstrated. This is hampered because both strain PICF7 and *V. dahliae* share the same niche (roots). Spatial separation of the pathogen and the BCA is needed to prove ISR. Our previous works have aimed to assess the effectiveness of systemic defense responses mediated by PICF7 against another olive pathogen affecting above-ground organs (*P. savastanoi* pv. savastanoi; Psv) and producing olive knot disease (Ramos et al., 2012). However, root colonization by PICF7 did not impair development of tumors in Psv-inoculated olive stems (Maldonado-González et al., 2013). Thus, even though PICF7 triggers a wide range of systemic defense responses (Gómez-Lama Cabanás et al., 2014), they do not seem to be effective against Psv. *Arabidopsis* has been earlier used to prove the involvement of ISR against *V. dahliae* mediated by the BCA *Paenibacillus alvei* K165 (Tjamos et al., 2005). Moreover, *A. thaliana* has also served to prove that an endophytic strain (*P. fluorescens* FPT9601-T5) originating from tomato (*Solanum lycopersicum* Mill) is able to trigger systemic defense responses effective against *P. syringae* pv. tomato (Wang et al., 2005). Consequently, two different approaches were followed. On the one hand, to evaluate whether presence of PICF7 in *Arabidopsis* roots can control disease caused by the foliar necrotrophic fungus *B. cinerea*. On the other hand, to assess whether PICF7 biocontrol performance against VW was affected in *A. thaliana* mutants unable to trigger ISR (*myb72*) or SAR (*sid2*). In the first approach, spatial separation of the BCA and the pathogen is guaranteed particularly because no evidence of endophytic colonization of *Arabidopsis* tissues by PICF7 was obtained. Therefore, mechanisms such as competition and/or antagonism can be excluded in this case. Despite the fact that results varied between bioassays, PICF7 was able to significantly decrease symptoms caused by *B. cinerea* to the same extent as *P. fluorescens* WCS417r (**Table 5**), as previously demonstrated for this strain (Van der Ent et al., 2008). This suggests that an effective systemic defense response is induced by PICF7 when present on *Arabidopsis* roots, corroborating previous findings found in olive aerial tissues (Gómez-Lama Cabanás et al., 2014). In addition, the use of *Arabidopsis* mutants revealed that strain PICF7 has the capability to control *V. dahliae* in different *A. thaliana* genotypes, although VW suppression was more consistently observed in *sid2* plants (**Table 3**). This may suggest that low levels of SA may help to increase the biocontrol performance of PICF7. Nevertheless, results from these bioassays were not consistent enough and VW biocontrol in *Arabidopsis* by PICF7 may rely on mechanisms and/or abilities (i.e., root endophytic colonization) that are not operative in this host in contrast to olive (Prieto et al., 2009). Moreover, mechanisms other than induced resistance could also be involved in suppression of *V. dahliae* since in *Arabidopsis* mutants impaired in either ISR or SAR disease control was still observed.

An additional objective of this study was to evaluate whether the use of the study system here developed can facilitate the identification of bacterial traits involved in VW biocontrol by strain PICF7. The colonization and VWO biocontrol abilities of PICF7 mutants affected in swimming motility (ME424), Pvd production (ME589), *in vitro* growth delay in PDA (ME419), or Cys auxotrophy (ME1508) were previously analyzed in olive (Maldonado-González et al., 2015). Here we examined the behavior of these mutants in *A. thaliana* Col-0. Population sizes of mutants did not significantly differ from that of strain PICF7 but in one experiment, stressing the variability also scored in bioassays carried out with olive plants. Interestingly, the Cys auxotroph mutant ME1508 always displayed the lowest population sizes, a similar behavior found in olive. Regarding to biocontrol performance, swimming motility and Pvd production of strain PICF7 seemed not to be crucial for VW suppression in *A. thaliana*, as also found in olive (Maldonado-González et al., 2015). However, both ME419 and ME1508 mutants did not control VW in some of the experiments (**Table 4**), suggesting that the nutritional requirements affected in these mutants can play a role in both colonization and biocontrol. An important outcome is that, overall, the behavior of the PICF7 mutants was similar to that previously reported in olive.

We conclude that the model plant *A. thaliana* provides a suitable and complementary approach to study *P. fluorescens* PICF7 traits involved in biocontrol of *V. dahliae*. In *Arabidopsis* the D and ND pathotypes of the pathogen showed a behavior similar to that in olive. PICF7 colonizes and persists in the *Arabidopsis* rhizosphere, and it decreases VW symptoms in this model plant. Moreover, the behavior of four selected PICF7 mutants affected in different traits was similar to that previously demonstrated in olive. These findings encourage the use of *A. thaliana* both for pathogenicity and virulence assessment of *V. dahliae* isolates and for the evaluation of large numbers of PICF7 mutant phenotypes related with biological control, saving time and space. In contrast, since we have not been able to demonstrate endophytism of strain PICF7 in *A. thaliana*, bacterial traits involved in this lifestyle cannot be evaluated in this plant. However, the different behavior that PICF7 displays in olive and *Arabidopsis* offers good opportunities to unravel mechanisms underlying endophytism by this bacterium.
